# An adaptive permutation approach for genome-wide association study: evaluation and recommendations for use

**DOI:** 10.1186/1756-0381-7-9

**Published:** 2014-06-14

**Authors:** Ronglin Che, John R Jack, Alison A Motsinger-Reif, Chad C Brown

**Affiliations:** 1 Bioinformatics Research Center, Department of Statistics, North Carolina State University, Raleigh, NC 27695, USA

## Abstract

**Background:**

Permutation testing is a robust and popular approach for significance testing in genomic research, which has the broad advantage of estimating significance non-parametrically, thereby safe guarding against inflated type I error rates. However, the computational efficiency remains a challenging issue that limits its wide application, particularly in genome-wide association studies (GWAS). Because of this, adaptive permutation strategies can be employed to make permutation approaches feasible. While these approaches have been used in practice, there is little research into the statistical properties of these approaches, and little guidance into the proper application of such a strategy for accurate p-value estimation at the GWAS level.

**Methods:**

In this work, we advocate an adaptive permutation procedure that is statistically valid as well as computationally feasible in GWAS. We perform extensive simulation experiments to evaluate the robustness of the approach to violations of modeling assumptions and compare the power of the adaptive approach versus standard approaches. We also evaluate the parameter choices in implementing the adaptive permutation approach to provide guidance on proper implementation in real studies. Additionally, we provide an example of the application of adaptive permutation testing on real data.

**Results:**

The results provide sufficient evidence that the adaptive test is robust to violations of modeling assumptions. In addition, even when modeling assumptions are correct, the power achieved by adaptive permutation is identical to the parametric approach over a range of significance thresholds and effect sizes under the alternative. A framework for proper implementation of the adaptive procedure is also generated.

**Conclusions:**

While the adaptive permutation approach presented here is not novel, the current study provides evidence of the validity of the approach, and importantly provides guidance on the proper implementation of such a strategy. Additionally, tools are made available to aid investigators in implementing these approaches.

## Background

Permutation testing is a popular nonparametric (distribution-free) randomization procedure, which provides a robust and powerful method of testing statistical hypothesis. In the classical form of the permutation test, the response is shuffled *b* times, and the test statistics are recorded for each permuted data set. These test statistics are then used to generate the distribution under the null hypothesis of no true association. If the observed test statistic is the *R*th largest among all the test statistics, then a *p*-value is declared as *R*/*b,* and the null hypothesis is rejected at this significance level [1-3].

A permutation test is commonly used when the standard distributional assumptions are violated [3]. For instance, quantitative trait loci (QTL) analysis typically assumes that quantitative traits are normally distributed within each genotype [4]. If the model is correctly specified, a simple one-way ANOVA test is often powerful in this situation. In more complicated models, large sample asymptotic approximations to test statistics can often be derived. Nevertheless, in practice, it is often difficult to determine the appropriate asymptotic distribution or the sample size may not be large enough for asymptotic assumptions to hold, limiting the generality of this approach. In these situations, the permutation test has become an emerging attractive choice, in particular when the distribution is doubtful. Such issues are becoming increasingly important as the field considers uncommon and even rare variants, with the introduction of the new exome chips with fixed sample sizes, as rare/less common variant frequencies can impact distributional assumptions like small sample sizes. As imputation tools have improved (from a methods development point of view, and with an increased number of reference genomes available), it is likely that many genome-wide association studies performed on early generation technologies will be reevaluated with imputed genotypes that cover more common variants, and less common or even rare variants.

The rapid explosion of computing capabilities has greatly facilitated the use of permutation tests [2], particularly in genomic studies [4]. The recent explosion in genome-wide association studies (GWAS) has presented several statistical challenges, as well as unprecedented computational burdens [5]. Most of the current GWAS methods were developed for analyzing each genetic marker, often a single nucleotide polymorphism (SNP), individually. However, in most GWAS applications, analyzing a large number of markers is required. In these situations, it is important to control the experiment-wise error rate (EWER) [6]. Using Bonferroni adjustment with the estimated number of informative markers in the genome, a common threshold for establishing significance in GWAS is generally considered to be *p* < 5*e* - 8 or *p* < 1*e* - 8, for *α*_*e*_ = 0.05 and 0.01, respectively [7]. Due to the discreet nature of permutation *p*-values, at least 2*e*7 and 1*e*8 permutation samples would be required for every SNP just to achieve the correct type I error rate. Even more samples would be needed to estimate *p*-values accurately at this level. This computational burden limits the application of standard permutation testing in GWAS.

Much success has been achieved with efficient computational algorithms to make each individual permutation test faster [8-10]. Although all of these studies yield tremendous improvements in computing times, they still have difficulty estimating *p*-values less than 5*e -* 8 accurately in reasonable time, and are limited to case–control data. These studies all rely on improvements or approximations to standard permutation testing, where a predetermined number of test statistics are computed for each locus, regardless of locus significance. In order to accurately estimate genome-wide significance, this number is necessarily large. Many resources wasted on markers having low associations. In a large-scale GWAS, it is assumed that the vast majority of SNPs are non-causal/non-associated. Identifying these SNPs early in permutation testing could reduce the total number of permutation samples. Computational burden could then be reserved for a small subset of SNPs with high associations to phenotype. These ideas were captured in a sampling scheme originally described by Besag and Clifford [1].

In this study, we propose an adaptive permutation strategy based on this existing schema [1], and generalize it to genomic studies. Furthermore, we establish a concordance between the standard and adaptive permutation approaches. We also make recommendations for the appropriate number of permutation samples to use, in order to achieve a desired level of accuracy for both permutation procedures using distributional theory. We design a wide range of simulation scenarios and demonstrate the validity of adaptive permutation. It achieves good power, and controls the type I error as well as experiment-wise error rate very well. In addition, the adaptive permutation is much faster than the standard permutation and provides good estimates of *p*-values, and thus it is computationally feasible for GWAS. Finally, we briefly demonstrate this approach in a real data analysis in a GWAS in pharmacogenomics.

## Methods

### Relationship between standard and adaptive permutation

#### Standard permutation: binomial distribution

Here, *b* permutation test statistics are calculated, with each statistic having probability *p* of being larger than the observed test statistic. In this case, the number of successes is distributed as binomial:

$$R∼\mathrm{Bin}\left(b,p\right),$$

and the estimate of  is $\hat{p}=R/b.$

#### Adaptive permutation: censored negative binomial distribution

In the adaptive approach, permutation test statistics are sampled until either *r* of these statistics are larger than the observed statistic, or *b* total permutations are calculated with *R* total successes, where *R* < *r*. In this case, the total number of permutations is distributed according to the censored negative binomial distribution:

$$B∼\mathrm{truncNB}\left(r,p,b\right).$$

The estimate of *p* is given by:

(1) $$\hat{p}=\left\{\begin{array}{c} \frac{r}{B},\;\left|R=r,\right)\frac{R+1}{b+1},R<r. \end{array}\right)$$

These two permutation tests are similar, except they differ in their sampling distributions. The standard permutation fixes the total number of permutations *b*, and calculates the number of successes, *R*, estimating *p* as *R*/*b,* where the numerator *R* is a random variable. The adaptive permutation fixes the total number of successes, *r*, estimating *p* using Eq. 1, where the denominator *B* is a random variable if *R* = *r*, or the numerator *R* is a random variable otherwise. To ensure the permutation stops in a finite number, the adaptive permutation stops if *b* permutations have been conducted, but the number of successes is still less than *r*.

### Choice of parameters for both permutation approaches

It is useful to define the desired precision of *p*-value estimation as *c*, or the fraction of the significance threshold  that equals the standard error in estimation, when *p* = *α*:

(2) $$c=\mathrm{SE}\left(\hat{p}\right)/\alpha .$$

For example, if *c* =0.1, and *α* =0.05, then we would choose permutation sampling parameters that guarantee the standard error in estimating *p*-values at 0.05 be less than *cα* = 0.005.

Choice for the total number of permutations determines the precision in estimating the *p*-value for traditional permutation testing. Here we have:

$$\mathrm{Var}\left(\hat{p}\right)=\alpha *\left(1-\alpha \right)/b.$$

Establishing the precision defined in Eq. 2 for statistically significant associations, and solving affords *b* = (1 - *α*)/*c*^2^*α*. Since *b* is quite large for small *α*, the central limit theorem provides that $\hat{p}$ is very close to being normally distributed. This implies that when *b* is chosen as (1 - *α*)/*c*^2^*α*, we have that $\left(\hat{p}-\mathrm{SE}\left(\hat{p}\right),\hat{p}+\mathrm{SE}\left(\hat{p}\right)\right)$ is approximately a 68% confidence interval (CI). This CI implies $P\left(\left(1-c\right)\alpha <\hat{p}<\left(1+c\right)\alpha \right)\approx 0.68$.

Similarly, choice of the censoring point, *b*, and the total number of successes, *r*, determines the precision in estimating the *p*-value for adaptive permutation testing. One way to ensure the same precision for the adaptive approach as the traditional approach is choosing *r* such that $P\left(\left(1-c\right)\alpha <\hat{p}<\left(1+c\right)\alpha \right)\approx 0.68$, which is guaranteed when $P\left(\hat{p}<\left(1-c\right)\alpha \right)=P\left(\hat{p}>\left(1+c\right)\alpha \right)\leq 0.16$. The smallest value of *r* that affords both of these probabilities can be calculated exactly using the qnbinom() function in R. These values for *r* (which are a function of *α* and *c*) were used in all simulations. The censoring point, *b*, was chosen to equal the total number of tests (also *b*) in the traditional approach. In this way, the adaptive permutation approach uses a censored negative binomial distribution. However, since the truncation occurs at *b* which is extremely large for small *α*, it approximates a (non-censored) negative binomial distribution quite well.

Point-wise error rate (PWER) is type I error rate for an individual test or the probability of incorrectly rejecting null hypothesis. Experiment-wise error rate (EWER), also called family-wise error rate (FWER), is the probability of making at least one type I error when performing a large number of related tests. Keeping EWER (*α*_*e*_) at a nominal significance level, the adjusted PWER was denoted as *α*_*p*_[6]. Since we assume all the tests are independent, it is appropriate to apply the Bonferroni correction approach, leading to *α*_*p*_ = *α*_*e*_/*m*, where *m* is the number of tests.

Choice for threshold values *b* and *r* is therefore a function of the number of tests *m*, PWER *α*_*p*_ and precision level *c*. The corresponding R code is available from http://www4.stat.ncsu.edu/~motsinger/Lab_Website/Software.html. Table 1 shows the recommendation of threshold values *b* and *r* in a series of scenarios.

**Table 1:** Adaptive permutation recommendations

	***c***	**0.1**		**0.2**	
***m***	***α***_***p***_	***b***	***r***	***b***	***r***
1	0.05	1,900	115	475	34
5	0.01	9,900	120	2,475	36
50	1e-3	99,900	121	24,975	36
500	1e-4	999,900	121	249,975	36
1,000	5e-5	1,999,900	121	499,975	36
10,000	5e-6	19,999,900	121	4,999,975	36
100,000	5e-7	199,999,900	121	49,999,975	36
1,000,000	5e-8	1,999,999,900	121	499,999,975	36

Recommendation of the number of permutations (*b*) and cut-off value (*r*) varying the number of SNPs (*m*), PWER (*α*_*p*_) and precision level (*c*), with a fixed EWER (*α*_*e*_ = 0.05).

### The adaptive permutation algorithm

The adaptive permutation algorithm proceeds as following:

*Step 1*: Determine the EWER (*α*_*e*_) and the number of independent tests (*m*). Apply the Bonferroni correction method to calculate the adjusted PWER as *α*_*p*_ = *α*_*e*_/*m*.

*Step 2*: Decide the precision level *c*. Choose the maximum number of permutations as *b* and the cut-off value, *r*, in order to achieve *c* (see Table 1).

*Step 3*: Start with SNP 1 as *i* = 1. Calculate the test statistics for *i*th SNP as $u_{i}^{*}$. For each *j*th permutation, calculate the test statistics *u*_*ij*_ and compare with $u_{i}^{*}$. Let *R*_*i*_ denote the number of values *u*_*ij*_ exceeding $u_{i}^{*}$ (success). Stop as soon as *R*_*i*_ equals *r*, and estimate the *p*-value as *r*/*B*_*i*_, where *B*_*i*_ is the number of permutations to achieve *r* number of successes. However, if this has not occurred after *b* permutations, we stop in any case and declare a *p*-value of (*R*_*i*_ + 1)/(*b* + 1). In sum, the estimate of *p*-value for *i*th SNP is:

$${\hat{p}}_{l}=\left\{\begin{array}{c} \frac{r}{B_{i}},\left|R_{i}=r,\right)\frac{R_{i}+1}{b+1},R_{i}<r. \end{array}\right)$$

If ${\hat{p}}_{l}<\alpha_{p}$, reject the null hypothesis at the nominal significance level of *α*_*e*_.

*Step 4*: Repeat step 3 until to complete all SNPs, that is, *i* = *m*.

### Simulation design

Each single nucleotide polymorphism (SNP) was assumed to have two alleles *A* and *a*, corresponding to the three genotypes *AA*, *Aa* and *aa*. An additive genetic model was applied, and thus the genotype value *S* was coded as 0, 1 and 2, corresponding to the number of minor alleles. Let *p*_*a*_ denote the minor allele frequency (MAF). Each SNP was assumed to be under Hardy-Weinberg Equilibrium (HWE) [11]. The quantitative phenotype *Y* was generated according to

(3) Y=βS+∈,

where *β* reflected the effect size of SNP and ∈ was the random error. Also, *m* is the total number of SNPs in the data set, and *n* is the sample size. No linkage disequilibrium (LD) was considered in the current study.

### Simulation design 1: comparison of ANOVA, standard and adaptive permutation type I error rates

Simulation was used to compare ANOVA to the standard and adaptive permutation procedures. Each SNP was generated assuming HWE with a MAF of 0.1. The quantitative phenotype *Y* was calculated using Eq. 3, where the error term ∈ was generated using the normal distribution with mean 0 and standard deviation 1, or the Student's *t*-distribution with 5 degrees of freedom. For the null distribution, the effect size *β* is 0. The primary objective of this simulation was to test the type I error rates of ANOVA and both permutation testing methods under correct and incorrect modeling assumptions. 10,000 datasets were simulated and for each replicate, 200 samples were generated. Using a precision (c) of 0.1, *b* was set to 9,900, and *r* was set to 120 (Table 1).

### Simulation design 2: comparison of ANOVA and adaptive permutation under GWAS settings

#### Type I error rates

One million replicates were simulated to resemble one million SNPs. Each replicate was generated independently. To obtain the Bonferroni adjustment *p*-value (*α*_p_ = 5e ‒ 8) and a *c* precision level of 0.1, the number of permutations is 1,999,999,900 and the cut-off value for adaptive permutation is 121. Data sets were generated under the null model, with a sample size of 200, SNP MAF = 0.1, and with error terms distributed as Student's *t* with 5 degrees of freedom. The sheer computational burden did not allow for comparison with standard permutation testing in this simulation.

#### EWER

To demonstrate the performance of ANOVA and adaptive permutation in terms of type I error rate and experiment-wise error rate (EWER), data sets of either 1,000 or 10,000 SNPs were generated. Each scenario was replicated 100 times, under the null with error terms following a Student’s *t*-distribution with 5 degrees of freedom. The means and standard deviations of the type I error rate across 100 replicates were calculated. Using a Bonferroni correction, with a desired EWER of 0.05, significance thresholds were set to 5*e* – 5 and 5*e* – 6 for 1,000 and 10,000 SNPs, respectively. EWER was calculated as the proportion of tests (across the 100 replicates) that have at least one false positive for any SNP. A sample size of 200 and MAF of 0.1 were used in this simulation.

#### Power

*β* represents the effect sizes for disease associated SNP(s). Varying effect sizes were used to ensure that the expected power, using ANOVA, was constant near 0.3, for various *α* levels. Based on the recommendations from Table 1, and a 0.1 precision level, 900, 1,900, 9,900 and 99,900 permutations were chosen for the *α* levels of 0.1, 0.05, 0.01 and 0.001, respectively. A single SNP with MAF of 0.1 was considered, and 5,000 data sets were replicated with a sample size of 500 for each. The power was defined as the proportion of times the null hypothesis was (correctly) rejected. Because ANOVA did not have the correct type I error rate when error terms were distributed using the Student's *t*-distribution, only the normal distribution was used for power comparisons.

### Simulation design 3: comparison of standard and adaptive permutation computation times

#### Permutation time under the null model

In this simulation, the computation times for the two permutation methods were compared. For the adaptive permutation, 1,000, 10,000 and 100,000 SNPs were simulated with 100 replicates for each scenario, while 1,000,000 SNPs were simulated with 3 replicates because of the burden of computation time. All *α* values were set according to Bonferroni correction level. The elapsed time was recorded using the proc.time() function in R, and the means and standard deviations of empirical elapsed time were calculated among all replicates. For the standard permutation, it was computationally infeasible to obtain the empirical time for even 1000 SNPs. However, using linear regression with a smaller number of SNPs, computation time was found to be almost exactly proportional to the number of calculated test statistics, which was the number of SNPs (*m*) multiplied by the number of permutations (*b*),

(4) time=γmb,

giving a model *r*-squared of 0.968. In this way, computation times for 1,000, 10,000, 100,000 and 1,000,000 SNPs could be estimated using extrapolation.

#### Relationship between time and effect size

Because of the nature of the adaptive permutation design, estimation of *p*-values for markers having a strong association with response is expected to take longer. To illustrate how the strength of association influences computation times, data were simulated using Eq. 3 with various effect sizes *β* ranging from 0 (null) to 1.8. For each effect size, 1,000 replicates were simulated using a MAF of 0.1 and a sample size of 200. The number of permutations, *b*, was set to 9,900. For standard permutation, the computation time was expected to be only dependent on the number of SNPs (*m*) and the number of permutations (*b*). For adaptive permutation, it was expected that the time would increase as the effect size of SNP increases.

The computation time required to evaluate 10,000 SNPs using b = 99,900 permutations was also estimated, by varying effect sizes of SNPs. For the adaptive approach, this was found by averaging the actual computation times across 100 replicates, while computation time for the standard permutation approach was predicted using Eq. 4.

All the simulation experiments were performed in R (http://www.r-project.org) using the High Performance Computing (HPC) cluster resource (hpc.ncsu.edu) at North Carolina State University. The HPC is a IBM Blade Center Linux Cluster, 1053 dual Xeon compute nodes with Intel Xeon Processors (mix of single-, dual-, quad-, and six- core), with 2-4GB per core distributed memory. The data analyses were performed using SAS 9.3 (http://www.sas.com). Plots were generated using R and JMP 10 (http://www.jmp.com).

### Real data analysis: application of adaptive permutation testing to published GWAS

In [12], 29 chemotherapeutic pharmaceutical agents were studied in concentration response cytotoxic assays across 520 cell lines with ~2 million genetic markers. The original analysis was performed with a MANCOVA approach using the Multivariate Ancova Genome-Wide Association Software (MAGWAS) package. We have extended the MAGWAS package using our adaptive permutation procedure. The following parameter choices were used: precision c = .2 and EWER (*α*_*e*_) = 5e-6. The entire set of SNP data was split by chromosome and separate processes were carried across all 22 chromosomes in parallel on a computing cluster with Xeon processors.

## Results

### Simulation result 1: comparison of ANOVA, standard and adaptive permutation type I error rates

**Figure 1:**
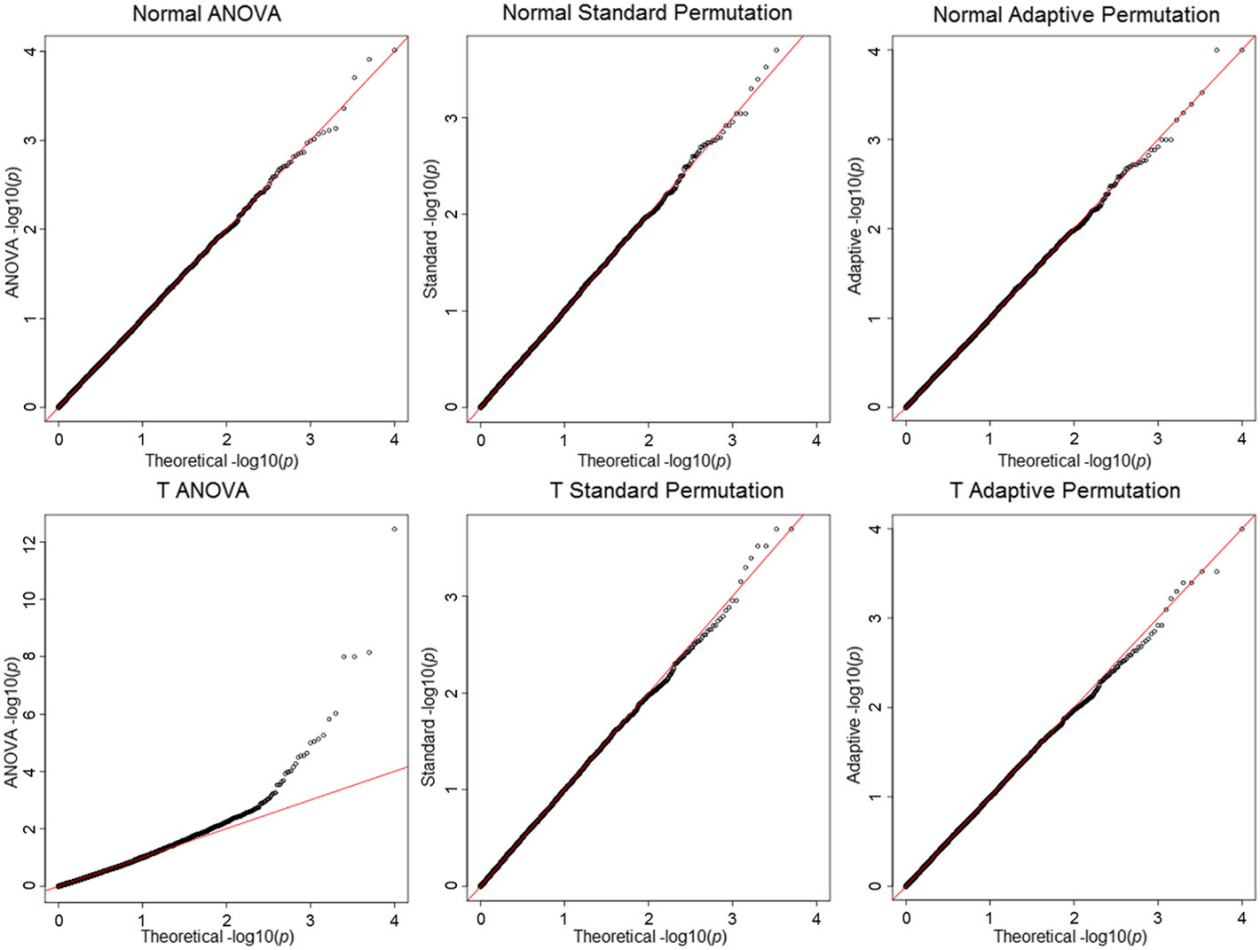
**Quantile-quantile plots for ANOVA, standard and adaptive permutation under the normal and the Student's *t*-distribution (df = 5) null model, with 10,000 replications and 9,900 permutations.**

Figure 1 shows the quartile-quartile (QQ) plots of the *p*-values from the ANOVA, standard and adaptive permutation tests under the normal and Student's *t*-distribution. It is expected all the tests should lie in a diagonal line under the null distribution. The upper three panels demonstrate all the three approaches have the correct type I error rate under correct modeling assumptions (normality). The bottom three panels show that ANOVA has an inflated type I error under the Student's *t*-distribution, especially for *p*-values smaller than 0.01, while both permutation tests are still valid.

Additional file 1: Figure S1 demonstrates that three approaches are similar when the model is correctly specified, while ANOVA has much smaller -values than permutation tests when the model is misspecified. Furthermore, it shows that the standard and adaptive permutation approaches provide similar *p*-values regardless if the model is specified correctly.

### Simulation result 2: comparison of ANOVA and adaptive permutation under GWAS settings

#### Type I error rates

**Figure 2:**
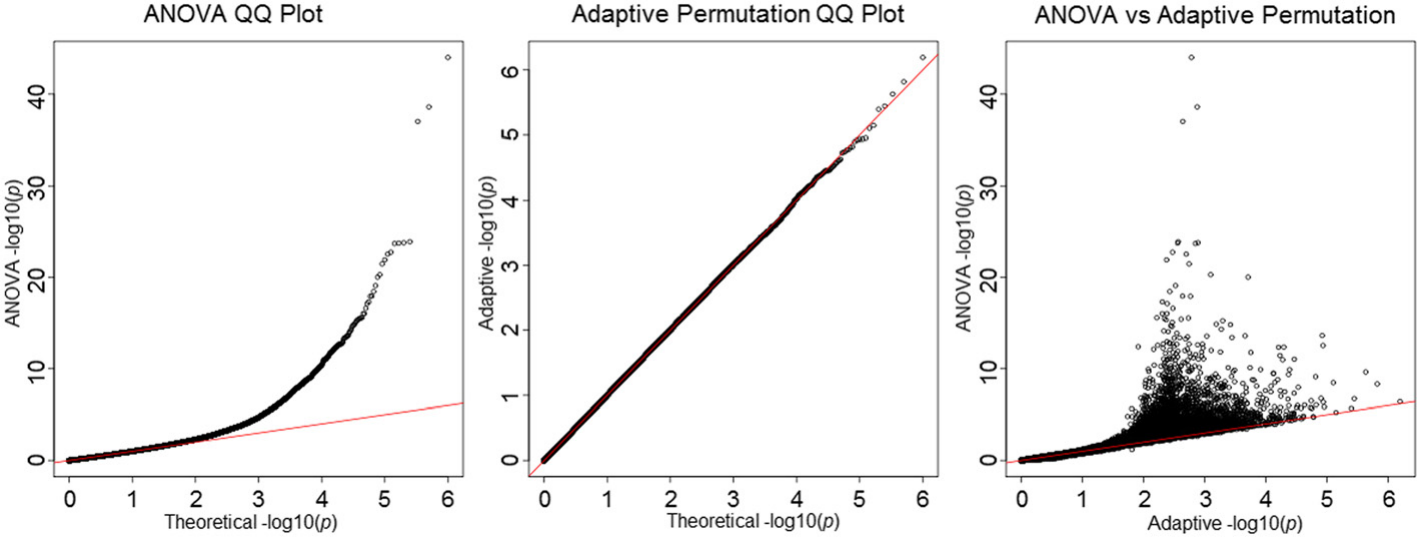
Quartile-quartile plots and comparison for ANOVA and adaptive permutation under the Student's t-distribution (df = 5) null model, with 1,000,000 replications and 1,999,999,900 permutations.

In Figure 2, the QQ plot of the ANOVA shows an obvious deviation from the expected line, in particular for small *p*-values. Adaptive permutation clearly portrays the validity under the null, for a large number of SNPs. Pairwise comparison of *p*-values between ANOVA and adaptive permutation demonstrates that the type I error rates for ANOVA are highly inflated.

#### EWER

The results of type I error rate for the 100 replicates are plotted in Additional file 1: Figure S2, for 1,000 and 10,000 SNPs respectively. For α_e_ = 0.05, the mean of type I error rate for ANOVA is around 0.055 and that for adaptive permutation is approximately 0.050, as shown in Table 2. A paired *t*-test shows that the ANOVA *p*-values are significantly larger than adaptive permutation *p*-values (*p* < 0.0001).

**Table 2:** Error rates

	**Means of type I error rate (SD)**			**EWER**	
***M***	**ANOVA**	**Adaptive**	*P*-value*	**ANOVA**	**Adaptive**
1,000	0.055 (0.0083)	0.050 (0.0081)	<0.0001	0.71	0.050
10,000	0.055 (0.0023)	0.050 (0.0020)	<0.0001	1.0	0.060

*A paired *t*-test was used to test the difference of type I error rate between ANOVA and adaptive permutation across all replicates.

Means of type I error rate and EWER comparison of ANOVA and adaptive permutation across 100 replicates, under the Student's *t*-distribution null model.

EWERs of ANOVA are 0.71 for 1,000 SNPs and 1.0 for 10,000 SNPs, while that of adaptive permutation are 0.05 and 0.06 respectively. It is clear that under the null model using the Student's *t*-distribution, ANOVA tests are too liberal and give very high EWERs. Adaptive permutation performs very well in controlling the EWER.

#### Power

Since we have already shown that adaptive permutation is good in controlling both PWER and EWER under the null model, the focus is to demonstrate there is no loss of power for permutation under the true model. As illustrated in Table 3, ANOVA and adaptive permutation provide identical power when the error terms are normally distributed. This demonstrates that ANOVA, outside of a mild computational benefit, has little advantage over adaptive permutation, even when modeling assumptions are correct.

**Table 3:** Power comparisons

				**Power (SE)**	
***α***_***p***_	***b***	***r***	***β***	**ANOVA**	**Adaptive**
0.1	900	108	0.21	0.297 (0.0064)	0.298 (0.0065)
0.05	1,900	115	0.27	0.289 (0.0064)	0.290 (0.0064)
0.01	9,900	120	0.37	0.284 (0.0064)	0.283 (0.0064)
0.001	99,900	121	0.49	0.278 (0.0063)	0.280 (0.0064)

Power comparison of ANOVA and adaptive permutation across 5,000 replicates, under the normal distribution true model.

### Simulation result 3: comparison of standard and adaptive permutation computation times

#### Permutation time under the null model

The predicted computation time is plotted in Table 4. Most notably, the adaptive permutation method computes one million SNPs faster than the standard method computes one thousand. Even at one million SNPs, the adaptive approach is computationally feasible, with an completion in 1.9 days, while standard permutation becomes infeasible even with just 10,000 SNPs, requiring an estimated 10 months.

**Table 4:** Computation times

			**Time**	
***m***	***α***_***p***_	***b***	**Standard predicted**	**Adaptive empirical (SD)**
1,000	5e-5	1,999,900	3.1 days	0.042 (0.026) hours
10,000	5e-6	19,999,900	10 months	0.55 (0.29) hours
100,000	5e-7	199,999,900	84 years	6.0 (2.0) hours
1,000,000	5e-8	1,999,999,900	8400 years	1.9 (0.079) days*

*Based on three observations; others based on 100 replicates.

Computation time comparison of standard and adaptive permutation for varying number of SNPs (*m*) and times of permutation (*b*) under the null model.

Causal/associated SNPs add significantly to the computational burden in the adaptive approach. However, for a more realistic scenario with 1 million non-associated SNPs and 5 extremely strongly associated SNPs, if all the five causal SNPs require maximal permutation times, the worst computation time would be around 17 days (on a single processor).

#### Relationship between time and effect size

**Figure 3:**
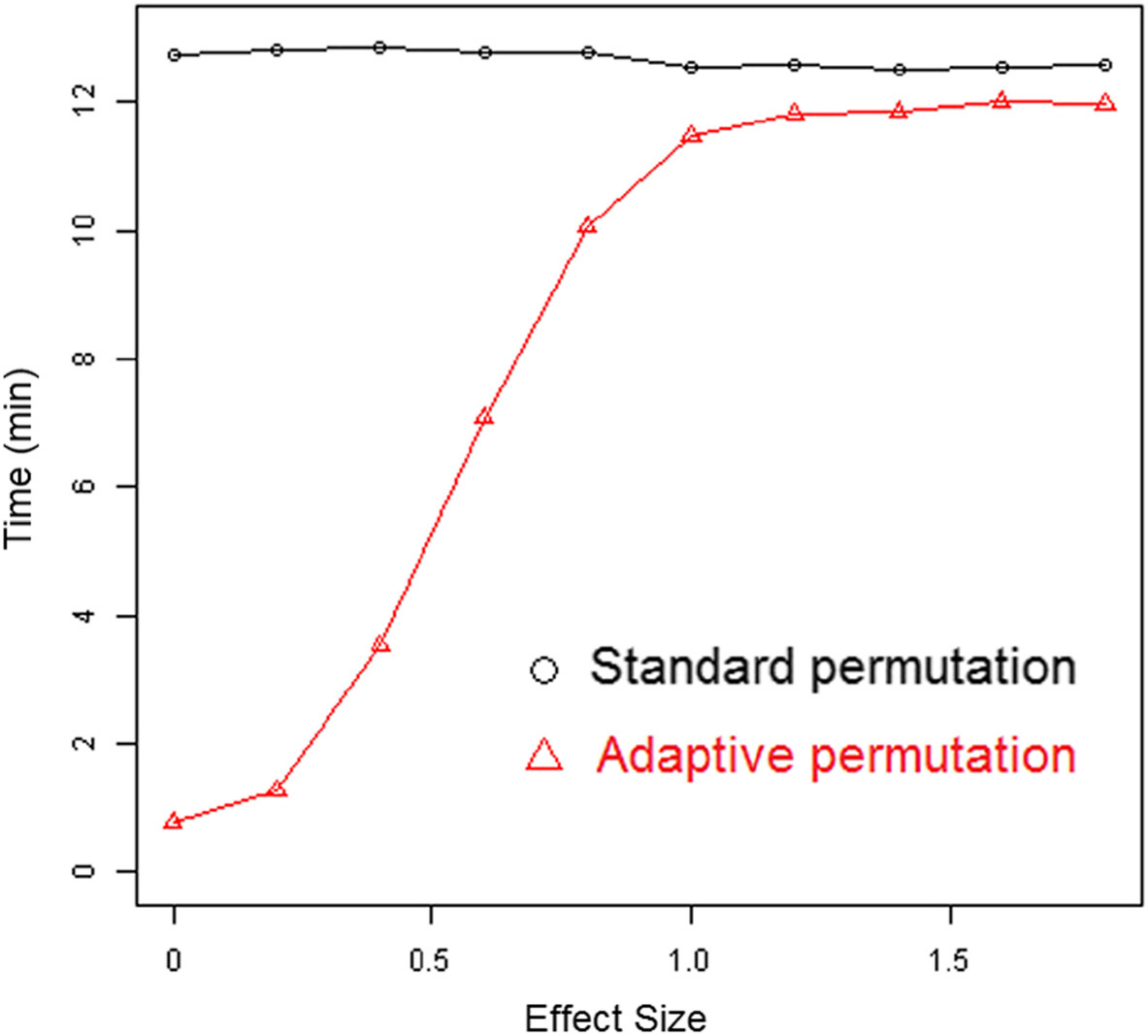
Computation time comparison of standard and adaptive permutation for varying effect size, with 1,000 replications and 9,900 permutations.

Another important consideration is the extent to which computational speed decreases as the strength of association between genotype and phenotype increases. Figure 3 shows that adaptive permutation is very sensitive to the effect size of SNP. When the effect size increases, the computation time of adaptive permutation increases in a non-linear way. When the effect size is large enough, the time would not change as the maximal permutation is attained. However, in regards to computation, standard permutation is constant to the effect size.

An additional study was conducted in a scenario of 10,000 SNPs and 99,900 permutations by varying effect sizes from weak to strong, with 100 replications for each. Additional file 1: Table S1 shows a similar pattern. For adaptive permutation, the computation time increases when the effect sizes of SNPs increase.

### Real data analysis

The adaptive permutation procedure on the drug, carboplatin, was compared with the previously published MANCOVA results. A pairwise comparison of negative log-transformed p-values across all SNPs (Additional file 1: Figure S3) shows the differences between the uncorrected and corrected association results. Since association analyses rely on large sample asymptotic theory, the previously published results reported associations solely on SNPs with at least 20 individuals for each genotype (black points), any SNPs in violation of this assumption (green or blue points) were previously filtered out. This filtering step was necessary to remove potentially spurious associations in the original analysis, and resulted in the removal of 38.4% of the SNPs from the original analysis. However, the adaptive permutation procedure was able to provide meaningful statistical tests for these SNPs, given the capacity to test for potential associations on rare or uncommon variants.

Overall, the top association (rs1982901, p < 10^-6^) from the original analysis remains significant after permutation testing. In the adaptive permutation analysis, an additional SNP crossed the significance threshold (rs6594545, p < 10^-6^). While not necessarily expected, this result does not contradict the conclusions of the original study, since the additional SNP is located physically close to the original top association. Thus, both associations are likely pointing to the same downstream biological mechanism.

## Discussion

We presented an adaptive permutation procedure for testing associations in high-dimensional studies having a large number of tests. This adaptive permutation advocates an intuitive idea that we may terminate the permutation at an early stage if there is little or even no evidence towards alternative based on our stopping rule, while perform exhaustive permutations for highly associated variables. This is a similar approach to that implemented in the popular software package PLINK (http://pngu.mgh.harvard.edu/~purcell/plink/), but other approaches do not provide guidance on the parameter choices to use when implementing such an adaptive approach.

By applying distributional theory, we provided some guidance for researchers about how to choose the number of permutations (*b*) and cut-off value (*r*), based on a series of factors, including the number of independent tests (*m*), the experiment-wise error rate (*α*_*e*_) and a desirable precision level (*c*). This guidance will be important for the proper application of our adaptive implementation, or others. The results tables provide parameter guidance for standard needs, and the available R code aids investigators in choosing parameters based on specifics of their own study.

Additionally, we show that the adaptive procedure maintains the correct type I and experiment-wise error rates, even when modeling assumptions are incorrect. However, there is essentially no loss in power when compared to the parametric approach. In addition, this adaptive procedure is computationally efficient enough to be applied to a large-scale GWAS. Indeed, this strategy has been successfully employed in a previous GWAS that involved mapping 520 individuals jointly across six responses, with several covariates, across over two million genetic markers. Albeit the study in question was not explicitly designed for locating rare variants, it is an important distinction that the adaptive permutation procedure gave meaningful results across all SNPs regardless of genotyping frequencies and without the need to filter SNPs based on any violation of asymptotic assumptions. All 22 chromosomes were run in parallel with the longest run time at 23.33 hrs. This is a remarkable example of how efficient this algorithm is, considering little effort was made to improve the computational efficiency of each individual permutation test. Also, parallel computations vastly improve the computational feasibility of the adaptive permutation procedure. Since each association test can theoretically be computed independently, the problem is embarrassingly parallel.

In the follow-up studies, it would be promising and attractive to incorporate some challenging issues, such as LD and multiple testing, in a more realistic simulation study. We suggest a very simple way to combine two component algorithms: the simple *M* method developed by Gao et.al [6] and our adaptive permutation algorithm. The simple *M* method was based on the Bonferroni correction, but it could account for composite LD correlation and derive an effective number of SNPs. By implementing this *M*_*eff* _ *G*_, it would be easy to replace the actual number of SNPs *m* to by the effective number of SNPs *M*_*eff* _ *G*_ in our algorithm step 1. Correspondingly, the adjusted PWER is *α*_*p*_ = *α*_*e*_/*M*_*eff* _ *G*_, leading to a less conservative threshold of point-wise significance and in turn a relatively small number of permutation times. It is believed that it may be highly computational efficient, while effectively accounting for the complex LD.

## Conclusions

In summary, the adaptive permutation procedure provides an easy, simple, fast and accurate test, and it is comparable to the existing permutation methods. While in conjunction with other multiple correction method, it is completely feasible to apply this adaptive method in a high-dimensional data, which we have shown through application to a published GWAS.

## Competing interests

The authors declare that they have no competing interests.

## Authors’ contributions

RC contributed to the study design, performed the simulation experiments, and drafted the manuscript. JRJ contributed to data analysis, extension of the MAGWAS software, and manuscript preparation. AMR contributed to the study design and manuscript preparation. CCB contributed to the study design and manuscript preparation. All authors read and approved the final manuscript.

## Supplementary Material

Additional file 1: Figure S1 Pair-wise comparisons for ANOVA, standard and adaptive permutation -log10(*p*) under normal and *t* (df=5) null model, with 10,000 replications and 9,900 permutations. **Figure S2**: Boxplots of type I error rate of ANOVA (0.055) and adaptive permutation (0.050), under the null *t*-distribution model (a paired *t*-test *p*<0.0001). **Figure S3**: Pairwise comparison of uncorrected (y-axis) and corrected (x-axis) negative log transformed p-values from a previously published GWAS. Each SNP (point) is colored according to genotype frequencies (AA, Aa, aa), where black denotes at least 20 individuals for all three possible genotypes. **Table S1**: Computation time (hours) comparison of standard and adaptive permutation for varying effect sizes, with 10,000 SNPs and 99,900 permutations, under the null model. Click here for file
