# Supplementary material for: An adaptive permutation approach for genome-wide association study: evaluation and recommendations for use
Source: BioData Min. 2014 Jun 14;7:9. doi: 10.1186/1756-0381-7-9 (PMC4070098; doi:10.1186/1756-0381-7-9)
Supplement: Additional file 1: Figure S1 — Pair-wise comparisons for ANOVA, standard and adaptive permutation -log10(p) under normal and t (df=5) null model, with 10,000 replications and 9,900 permutations. Figure S2: Boxplots of type I error rate of ANOVA (0.055) and adaptive permutation (0.050), under the null t-distribution model (a paired t-test p<0.0001). Figure S3: Pairwise comparison of uncorrected (y-axis) and corrected (x-axis) negative log transformed p-values from a previously published GWAS. Each SNP (point) is colored according to genotype frequencies (AA, Aa, aa), where black denotes at least 20 individuals for all three possible genotypes. Table S1: Computation time (hours) comparison of standard and adaptive permutation for varying effect sizes, with 10,000 SNPs and 99,900 permutations, under the null model. [file 1756-0381-7-9-S1.docx]

**Supplemental Information**


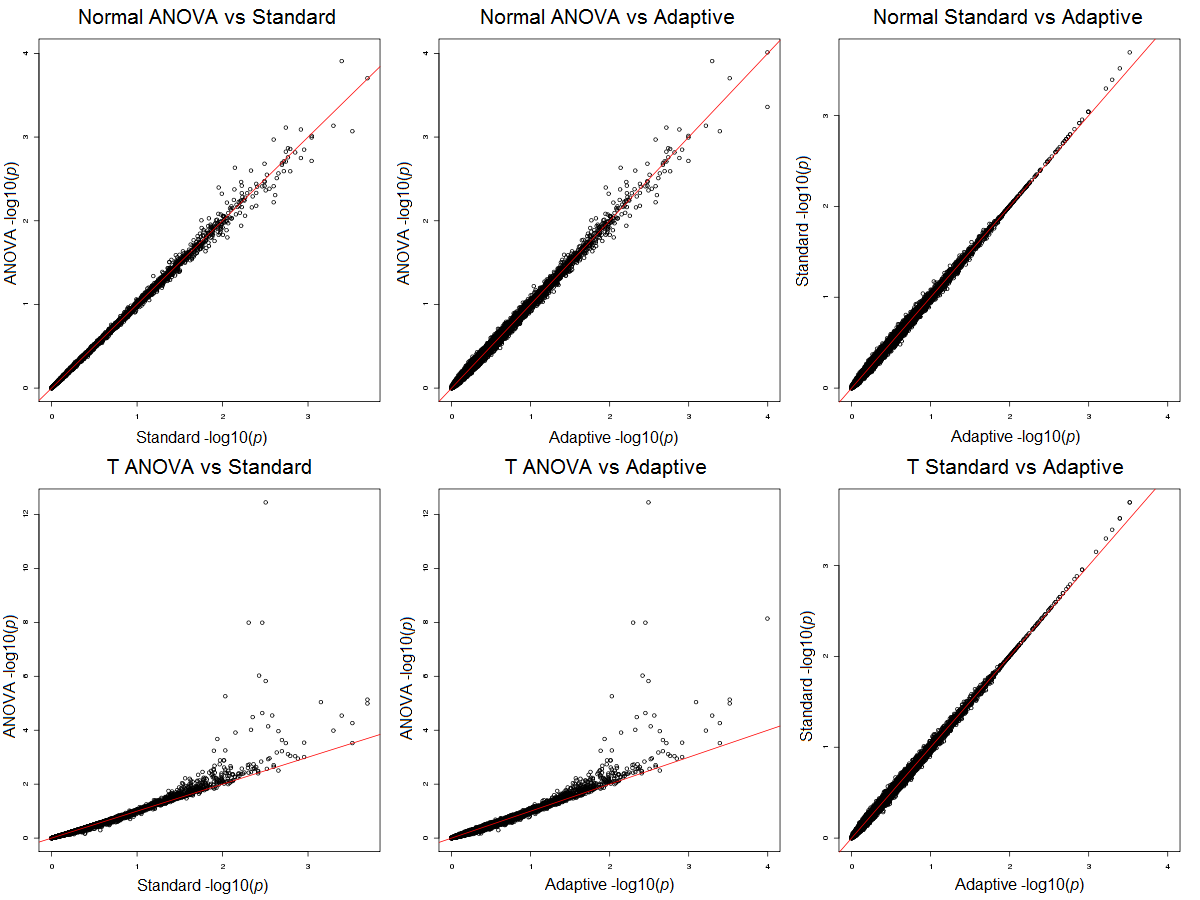


Figure S1: Pair-wise comparisons for ANOVA, standard and adaptive permutation -log10(*p*) under normal and *t* (df=5) null model, with 10,000 replications and 9,900 permutations.


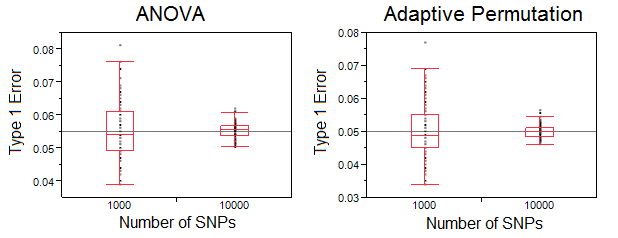


Figure S2: Boxplots of type I error rate of ANOVA (0.055) and adaptive permutation (0.050), under the null *t*-distribution model (a paired *t*-test *p*<0.0001).

Table S1: Computation time (hours) comparison of standard and adaptive permutation for varying effect sizes, with 10,000 SNPs and 99,900 permutations, under the null model.

|  | Time | |
| --- | --- | --- |
| Effect size ($\beta$) | Standard predicted | Adaptive empirical (SD) |
| No ($0$) | 37 | 0.33 (0.063) |
| Weak (0.20) | 37 | 0.71 (0.11) |
| Moderate (0.50) | 37 | 5.1 (0.44) |
| Strong (0.80) | 37 | 17 (2.2) |

Note: Adaptive permutation time is mean (standard deviation) of 100 replicates. Standard permutation time is predicted based on a multiplicative model (Equation 4).


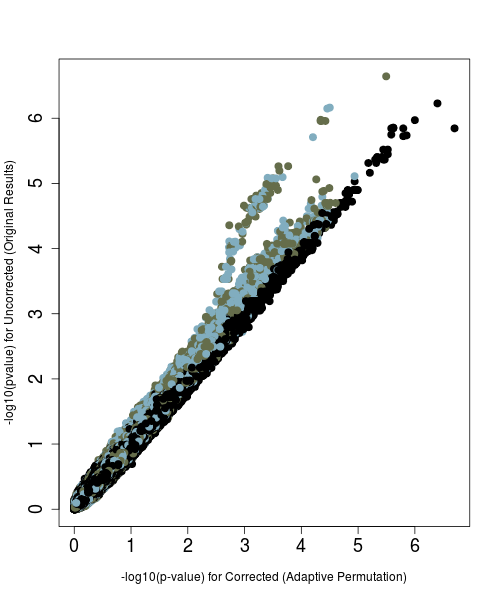


Figure S3: Pairwise comparison of uncorrected (y-axis) and corrected (x-axis) negative log transformed p-values from a previously published GWAS. Each SNP (point) is colored according to genotype frequencies (AA, Aa, aa), where black denotes at least 20 individuals for all three possible genotypes..
